# Increasing Width of Attached Gingiva Using Modified Apically Repositioned Flap Technique Combined With Platelet-Rich Fibrin: A Case Report

**DOI:** 10.7759/cureus.109542

**Published:** 2026-05-24

**Authors:** Mohanagowri N, Balakumaran Maroudaviran, Kennedy Babu, Soorya K.V

**Affiliations:** 1 Periodontics, Mahatma Gandhi Postgraduate Institute of Dental Sciences, Pondicherry, IND

**Keywords:** attached gingiva, gingival recession, modified apically repositioned flap (marf), periodontal health status, platelet rich fibrin (prf)

## Abstract

Adequate attached gingiva is cardinal for periodontal health maintenance. It helps to prevent apical migration of the gingival margin and serves as a stable barrier against plaque accumulation and plaque-induced inflammation of the gingiva. To increase attached gingiva, many techniques have been used. The modified apically repositioned flap (MARF) technique is considered one of the simplest methods to increase attached gingiva. In this technique, a partial-thickness flap is elevated using a single horizontal incision and then repositioned apically, which results in the exposure of the periosteum. This exposed periosteum may cause postoperative discomfort to the patient. To overcome this, platelet-rich fibrin (PRF) is placed over the exposed periosteum to promote healing and reduce postoperative discomfort. This case presentation addresses the amelioration of the width of the attached gingiva using MARF combined with PRF.

## Introduction

The keratinized gingiva consists of unattached gingiva, which is limited by the free gingival groove, and the attached gingiva, which extends from the groove to the mucogingival junction [1,2]. The primary function of attached gingiva is to defend the underlying structures of the periodontium, maintain the integrity of the gingival margin, and resist the pull of muscle fibers. Attached gingiva protects the periodontium from masticatory trauma and toothbrushing trauma [3]. Due to these protective functions of attached gingiva, evaluation of the width of attached gingiva is an important component of a comprehensive periodontal examination. Thus, clinical procedures to increase the apico-coronal dimension of attached gingiva are common in periodontics. Techniques used to ameliorate the width of keratinized tissue (WKT) are the apically repositioned flap, free gingival graft (FGG), and complete denudation and periosteal separation techniques. The most commonly used techniques are the apically repositioned flap and FGG.

In an apically repositioned flap, the mucoperiosteal flap is elevated and repositioned apically by two vertical incisions, and sutures are used to secure the flap apically. Healing is by secondary intention due to the presence of denuded bone, and it has the risk of bone resorption [4]. For augmenting the gingiva, FGG, which is widely used as the gold standard, has considerable disadvantages such as donor site complications, patient discomfort, and poor color match with surrounding tissues, which are neutralized in the MARF technique [2]. The technique involves a single horizontal incision to elevate the split-thickness flap, and the flap is moved apically and secured with sutures. Patients may develop postoperative discomfort due to exposure of the periosteum, so to overcome the patient's postoperative discomfort and promote wound healing, Platelet Rich Fibrin (PRF) is used to cover the periosteum [4].

PRF is a second-generation platelet concentrate used as an autologous biomaterial among various autologous grafts. It is a dense, elastic fibrin network that contains platelets, leukocytes, growth factors, and cytokines that promote tissue healing and regeneration. The alpha granules in the concentrated platelets in PRF secrete biomolecules, including cytokines, metalloproteinases, and growth factors. These components are vital for promoting tissue regeneration and healing [5-7]. Recent systematic reviews indicate that injectable PRF can modify gingival phenotype and improve soft-tissue outcomes, supporting its use as an adjunct in mucogingival procedures [8].

This clinical case presentation describes the use of a combination of PRF and MARF techniques to augment the attached gingiva.

## Case presentation

Patient history

A 21-year-old male patient presented with complaints of difficulty during toothbrushing and food debris accumulation in the lower anterior region. The patient did not have any adverse habits and had a satisfactory oral hygiene status. No relevant medical history was reported by the patient. 

Clinical Examination

**Figure 1:**
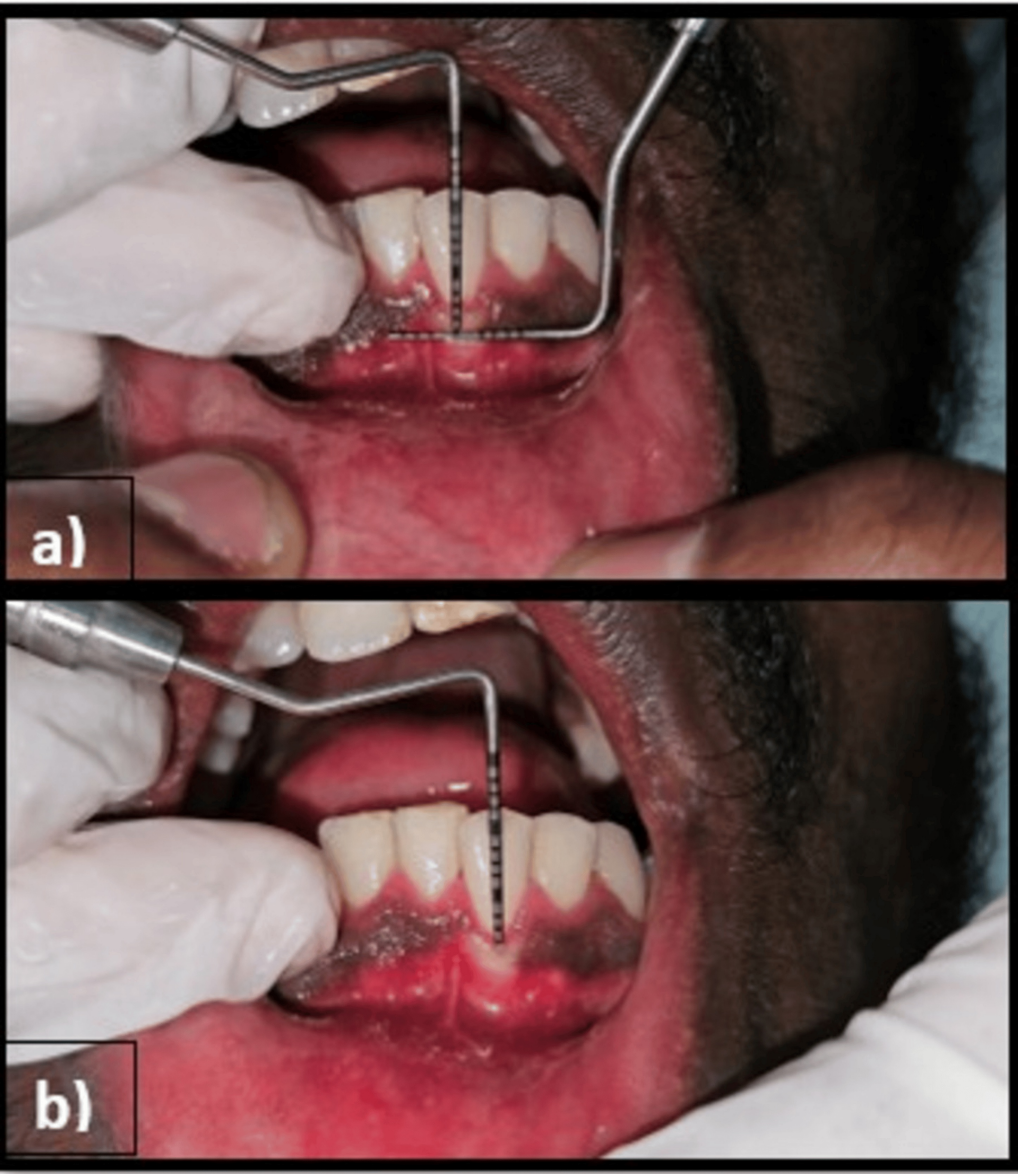
Pre-operative images a) measurement of keratinized gingiva b) probing depth Measurements were recorded using UNC-15 periodontal probe.

On examination, labially placed 31 with 1 mm of probing depth, 1.5 mm of keratinized gingiva, and 0.5 mm of attached gingiva (Figure 1), with a thin gingival phenotype was present.

Treatment

The treatment plan was explained to the patient, and informed consent was obtained. Routine blood investigations were performed before initiating Phase I therapy to assess the patient’s systemic health status. The recorded values were hemoglobin (Hb) 12 g/dL (reference range: 12-16 g/dL), total leukocyte count (TLC) 6,900 cells/µL (reference range: 4,000-11,000 cells/µL), bleeding time (BT) 3 minutes, 10 seconds (reference range: 2-7 minutes), clotting time (CT) 5 minutes 55 seconds (reference range: 5-11 minutes), and random blood sugar (RBS) 109 mg/dL (reference range: 70-140 mg/dL). All hematological parameters were within normal physiological limits. Phase I therapy, including scaling and root planing, was done, and oral hygiene instructions were given. After four weeks of the maintenance phase, the patient was reviewed and planned for phase II therapy.

The surgical technique MARF was performed based on the protocol developed by Carnio et al. [9]. After administration of local anesthesia, a horizontal incision was made with a BP blade number 15c. An incision was placed approximately 0.5 mm coronal to the mucogingival junction, ensuring that this narrow band of tissue remained with the flap. The horizontal incision was extended mesiodistally till the mid-buccal area of the adjacent teeth to prevent the need for a vertical releasing incision.

**Figure 2:**
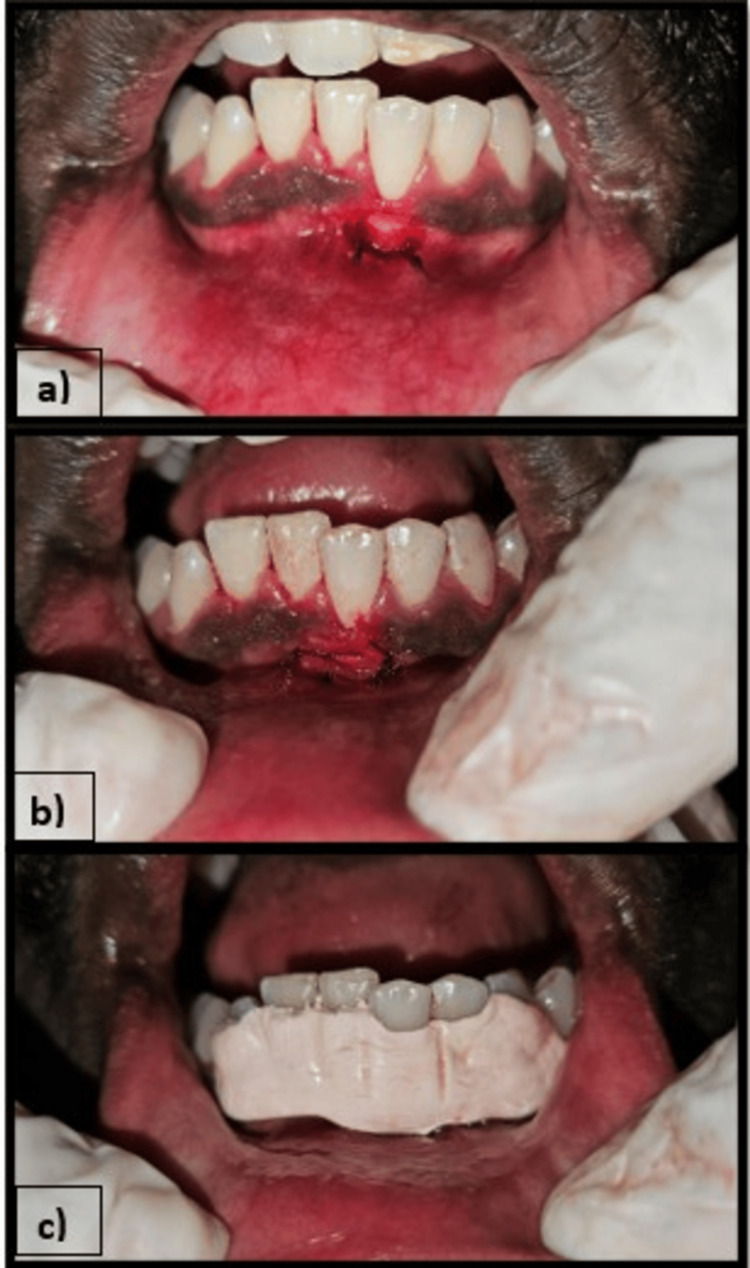
Surgical procedure a) split thickness flap elevated and positioned apically b)PRF placed and secured with 4-0 vicryl sutures c) Periodontal dressing in place

A partial thickness flap was carefully elevated, extending about 4 mm in the apical direction, and the apically repositioned flap was secured by placing simple interrupted sutures using 4-0 Vicryl directly to the underlying periosteum. This technique leaves a zone of periosteum exposed between the newly positioned flap and the original gingival margin. PRF is placed over the exposed periosteum and sutured using 4-0 Vicryl. A coe-pak was placed to secure the surgical area during initial healing (Figure 2).

Preparation of PRF

To prepare PRF, about 10 ml of whole blood was obtained from the patient via venipuncture at the antecubital fossa, and the collected blood was poured into the tube without an anticoagulant for PRF preparation. Then, immediately, the tube was centrifuged for 10 minutes at 3000 rpm at room temperature. The structured fibrin clot (PRF) formed in the center of the tube, just in the middle of the red corpuscle at the base, and the acellular plasma (PPP) at the top.

Postoperative Care

Paracetamol 500 mg and amoxicillin 500 mg TID were prescribed to the patient, and postsurgical instructions were given. A 0.2% chlorhexidine mouthwash was prescribed to the patient, and they were instructed to use it twice every day for two weeks. The patient was advised not to brush the area for two weeks. The Coe pack and sutures were removed after a period of two weeks. Oral hygiene instructions were reinforced.

Follow-Up

Healing was satisfactory, and the patient reported minimal pain and discomfort at the first follow-up at two weeks. The tissue at the operative site exhibited an excellent color match with its surroundings. Table 1 shows comparative measurements at baseline and follow-ups.

**Table 1:** Comparative measurements at baseline and follow-ups with respect to 31

Time line	wKT	PD	WAG
Baseline	1.5mm	1mm	0.5mm
1 month	3mm	1mm	2mm
6 months	3mm	1mm	2mm

**Figure 3:**
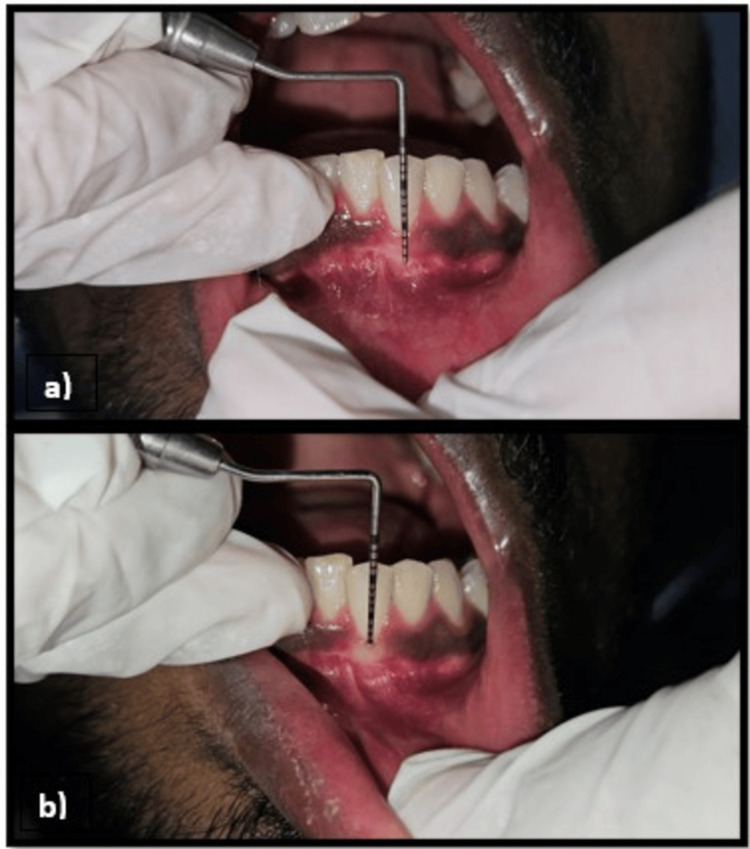
Postoperative six months a) measurement of keratinized gingiva b) probing depth Measurements were recorded using a UNC-15 periodontal probe.

Results were also consistent at the final follow-up at six months (Figure 3).

## Discussion

Inadequate width of attached gingiva causes plaque accumulation and increases the rate of progression of attachment loss. Patients will also have discomfort while tooth brushing when the width of the attached gingiva is inadequate. Therefore, to maintain proper health of the periodontium, an adequate amount of attached gingiva is required [10]. Wennstrom and Lindhe stated that 2 mm of keratinized gingiva is sufficient to maintain healthy gingiva [11]. Lang and Loe did a study in 1972; they found that areas with less than 2 mm of keratinized gingiva persisted in remaining inflamed in spite of adequate oral hygiene [1]. Areas with less than 2 mm of attached gingiva should be carefully monitored for attachment loss.

The most common technique to augment the attached gingiva is the apically repositioned flap and FGG. Friedman first introduced the apically repositioned flap technique [12]; however, it has been associated with an increased risk of bone resorption. Björn introduced the free gingival graft technique, which has disadvantages such as donor site morbidity, patient discomfort, and poor color match with surrounding tissues [13]. To overcome these disadvantages, Carnio and Miller proposed the modified apically repositioned flap (MARF) technique to augment the keratinized gingiva [9]. The rationale for MARF is to augment the attached gingiva by minimal surgical intervention and to match the color of the augmented attached gingiva with adjacent tissue [2]. In the MARF technique, a single horizontal incision is used to elevate a partial-thickness flap, which is displaced apically, after which it is approximated with sutures. This results in periosteum exposure to the oral cavity, allowing the granulation tissue to develop over the wound and eventually mature into keratinized tissue. Since the exposed periosteum may lead to postoperative pain and discomfort, a PRF membrane is placed over the exposed periosteum to reduce postoperative discomfort and promote faster healing [4]. Comparative trials of injectable PRF versus other soft-tissue modifiers provide context for expected phenotype changes and measurement approaches [14].

The purpose of placing a PRF membrane over the exposed periodontium in the MARF technique is to promote faster wound healing by releasing growth factors and help preserve the underlying bone, preventing its remodeling. PRF acts as a natural dressing. Post-operative usage of chlorhexidine mouthwash has an undesirable impact on PRF cells. To overcome the drawback, a periodontal dressing (Coe pack) is placed over the surgical site [4].

The granulation cells that populate the exposed periosteum determine the type of new tissue formation [11]. In MARF, the operative site is enclosed by keratinized tissue, which interferes with the nonkeratinized epithelial cell migration originating in the oral mucosa. The epithelial membrane cells migrate from the margins of the operative site, enclosing the exposed periosteum. In this case report, a two-week postoperative follow-up showed healthy granulation tissue over the surgical site, and the patient experienced a minimal amount of discomfort and pain. The treated area exhibited a resemblance to the adjacent tissue. Healing was satisfactory. Color match and consistency were excellent at one-month follow-up and consistent at six-month follow-up. Also, at the end of six months of PD, the width of KT and the width of attached gingiva were 1 mm, 3 mm, and 2 mm, respectively, and the width of attached gingiva and the width of KT increased from 0.5 mm to 2 mm and from 1.5 mm to 3 mm from baseline to six months, respectively.

Carnio and Camargo conducted a long-term retrospective study, which concluded that the MARF method is a reliable and predictable method to augment the region of keratinized tissue as well as the attached gingiva in the apico-coronal dimension, without the loss of attachment over long periods [15].

A prerequisite of 0.5 mm of attached gingiva preoperatively is a major disadvantage of the modified apically repositioned flap method. This mandate ensures the full perimeter of the wound is surrounded by keratinized tissue, which is crucial for the granulation tissue formation during the healing of the wound. A contraindication for performing the MARF technique is the presence of bone dehiscence, as it may cause complications such as marginal bone loss and gingival tissue [2,12].

Carnio et al. did a long-term retrospective case series study using the MARF technique in an area with no keratinized tissue to create attached gingiva, which resulted triumphantly in ameliorated attached gingiva without loss of attachment [16].

## Conclusions

In this case, MARF combined with PRF was associated with an increase in the width of attached gingiva and minimal postoperative discomfort. However, as this is a single case report, further controlled clinical studies are necessary to confirm the efficacy, predictability, and long-term stability of this approach. The MARF technique is a modest technique to augment the width of attached gingiva compared to other mucogingival surgical procedures. The technique is less invasive than FGG and apical repositioning flaps. Incorporating PRF with the MARF technique increased the width of the attached gingiva as well as reduced pain and patient discomfort postoperatively. The biological properties of PRF accelerated the healing process by providing a scaffold for tissue regeneration over the exposed periosteal bed. This combined approach offers a predictable and patient-friendly treatment option for cases requiring augmentation of keratinized tissue. The technique can be particularly beneficial in patients with limited vestibular depth and inadequate zones of attached gingiva. Further long-term follow-up studies would help establish the stability of the attached gingiva gained and validate the efficacy of this combined therapeutic approach.
